# Gallstone Ileus Presenting As Small Bowel Obstruction: A Surgical Case Report

**DOI:** 10.7759/cureus.88558

**Published:** 2025-07-22

**Authors:** Wilhelm Hansen, Mohammed Fayaz Kimmie, Usama Anjum, Azeemah Bulbulia

**Affiliations:** 1 General Surgery, University of the Witwatersrand, Johannesburg, Johannesburg, ZAF; 2 General Surgery, Robert Mangaliso Sobukwe Hospital, Kimberley, ZAF

**Keywords:** acute care surgery and trauma, bowel obstruction, colonic gallstone ileus, general surgery, giant gallstone

## Abstract

Gallstone ileus is an uncommon complication of cholelithiasis. This case report discusses an 83-year-old patient who presented with chronic, intermittent abdominal discomfort that progressed to persistent nausea and vomiting, necessitating hospital admission. A CT scan confirmed the diagnosis of gallstone ileus, demonstrating Rigler’s triad. The patient was successfully managed with a surgical enterotomy, specifically an enterolithotomy. Notably, the patient had no prior admissions for gallstone-related issues, despite a history of recurrent abdominal symptoms that had not been previously investigated. This case highlights the importance of early diagnosis and management of cholelithiasis, even in asymptomatic patients, to prevent delayed complications such as gallstone ileus, and underscores the critical role of imaging in the preoperative assessment of bowel obstruction.

## Introduction

Gallstone ileus is a rare but serious complication of cholelithiasis, accounting for approximately 1% to 4% of mechanical bowel obstruction cases in adults [1-4]. Cholelithiasis refers to the formation of gallstones, solid concretions composed of bile components such as cholesterol, bile pigments, and calcium salts, within the gallbladder [5]. It is commonly associated with risk factors such as advanced age, female sex, obesity, and rapid weight loss. In rare cases, cholelithiasis can lead to the development of a biliary-enteric fistula, allowing a gallstone to migrate into the gastrointestinal tract and cause obstruction [6]. This condition is known as gallstone ileus. The obstruction typically occurs at the ileum, where the lumen is narrowest. Diagnosis is most often made through imaging, with Rigler’s triad, pneumobilia (air within the biliary tree), small bowel obstruction, and an ectopic gallstone, being pathognomonic and most reliably identified on CT scans [1-4].

This condition develops through the formation of a fistulous tract between the gallbladder and the adjacent bowel, as illustrated in the present case. The literature describes this process as resulting from chronic inflammation, during which the gallbladder wall becomes eroded due to prolonged contact with the neighbouring bowel, most commonly the duodenum or small intestine, thereby facilitating stone migration [1].

Surgical intervention remains the cornerstone of treatment for gallstone ileus. Current literature supports single enterolithotomy as the preferred approach for stone extraction in high-risk patients [1]. However, the need for interval biliary surgery following the initial operation remains uncertain [4, 7]. Gallstone ileus is associated with a high mortality rate (reported between 12% and 27%), particularly in elderly patients, due to diagnostic delays and its often nonspecific presentation [7]. This emphasises the importance of early recognition and intervention.

We present the case of an elderly female who developed small bowel obstruction due to gallstone ileus, which was diagnosed radiologically and successfully managed surgically.

## Case presentation

An 83-year-old female with a known history of hypertension, managed on medication, and a documented penicillin allergy, presented with a one-day history of constipation and multiple episodes of vomiting. The vomiting was food-stained, non-bloody, and non-bilious. She also reported associated abdominal pain and mild distension, particularly in the epigastric region. Despite the constipation and vomiting, she continued to pass flatus. There was no history of fever, haematemesis, or melaena. She denied any recent dietary changes, travel, or use of over-the-counter medications.

She described occasional previous episodes of abdominal discomfort, although none had been severe enough to warrant medical evaluation. Notably, the patient also reported a six-month history of intermittent right upper quadrant discomfort, which had escalated to constant epigastric pain and vomiting over the preceding 24 hours. Her past surgical history included a Caesarean section and an appendicectomy, both performed more than twenty years ago; however, she was unable to recall the specific dates or indications. She had no known history of gallstones or biliary colic and had not previously undergone hepatobiliary imaging. Prior to admission, she was functionally independent, with no limitations in her daily activities.

On clinical assessment, the patient appeared alert and oriented but mildly dehydrated. Her vital signs were stable: heart rate of 96 beats per minute, blood pressure of 114/71 mmHg, respiratory rate of 19 breaths per minute, and oxygen saturation between 96% and 98% on room air. Her Glasgow Coma Score was 15/15. A nasogastric tube was inserted, draining bile-stained gastric contents.

Abdominal examination revealed epigastric tenderness without guarding or rebound tenderness. No masses were palpable, and abdominal distension was mild. Digital rectal examination revealed an empty rectum with normal sphincter tone and no palpable masses. Examination of the other systems was unremarkable.

Initial management in the emergency unit included intravenous fluid resuscitation, nasogastric decompression, and abdominal radiographs. The X-rays demonstrated dilated loops of small bowel and multiple air-fluid levels, consistent with small bowel obstruction. Following surgical referral, a CT scan of the abdomen was arranged for further evaluation.

Laboratory investigations on admission revealed Grade 1 hyponatraemia (sodium: 130-134 mmol/L) and elevated haemoglobin (18.4 g/dL) and haematocrit (0.556 L/L), consistent with haemoconcentration secondary to dehydration. Renal function was significantly impaired, with urea elevated to 14.5 mmol/L and creatinine initially unavailable. Post-resuscitation, urea decreased to 5.0 mmol/L, and creatinine normalised to 52 µmol/L, with an estimated glomerular filtration rate (eGFR) >60 mL/min/1.73m², confirming reversibility. Liver function tests showed elevated total bilirubin (34 µmol/L), direct bilirubin (6 µmol/L), and mildly raised gamma-glutamyl transferase (GGT) (42 U/L), suggesting biliary tract involvement, which aligned with imaging findings. These values improved postoperatively. Inflammatory markers were markedly raised preoperatively, with CRP peaking at 260 mg/L and WCC at 11.97 ×10⁹/L. CRP decreased to 69.2 mg/L postoperatively, consistent with resolution of inflammation. INR was elevated at 2.84, likely due to transient hepatic dysfunction or vitamin K depletion, and normalised to 1.5 thereafter. Platelet count dropped from 251 ×10⁹/L to 116 ×10⁹/L postoperatively, most likely due to haemodilution or mild consumptive coagulopathy, with gradual recovery observed (Table 1).

**Table 1:** Blood results.

Test	April 24, 2025	April 29, 2025	May 7, 2025	Reference Range
Sodium	134	130	133	135-145 mmol/L
Potassium	4.3	4.3	4.2	3.5-5.0 mmol/L
Chloride	86	89	92	98-106 mmol/L
Bicarbonate (Total CO₂)	20	25	31	22-29 mmol/L
Anion Gap	32	20	8	9-16 mmol/L
Urea	14.5	5	2.7	2.5-7.8 mmol/L
Creatinine	–	52	53	60-110 µmol/L
eGFR (MDRD)	–	>60	>60	>60 mL/min/1.73m²
Calcium	2.54	–	–	2.15-2.50 mmol/L
Magnesium	0.92	–	–	0.66-1.07 mmol/L
Phosphate	0.86	–	–	0.81-1.45 mmol/L
Albumin	40	–	31	35-50 g/L
Total Bilirubin	34	–	9	5-21 µmol/L
Direct Bilirubin	6	–	2	0-3 µmol/L
Alanine Aminotransferase (ALT)	20	–	27	5-40 U/L
Aspartate Aminotransferase (AST)	–	–	24	5-40 U/L
Alkaline Phosphatase (ALP)	72	–	65	30-120 U/L
Gamma-Glutamyl Transferase (GGT)	42	–	29	<40 U/L
Total Protein	–	–	50	64-83 g/L
C-Reactive Protein	–	228	37	<5.0 mg/L
White Cell Count	11.97	11.01	7.89	4.0-11.0 ×10⁹/L
Red Cell Count	6.38	5.29	4.88	3.93-5.40 ×10¹²/L
Haemoglobin	18.4	15.4	14.2	13.0-17.0 g/dL
Haematocrit	0.556	0.46	0.46	0.40-0.50 L/L
Mean Corpuscular Volume (MCV)	87.2	87	87	80-96 fL
Mean Corpuscular Haemoglobin (MCH)	28.9	29.2	29.2	26.1-33.5 pg
Mean Corpuscular Hb Concentration (MCHC)	33.1	33.5	33.5	32.7-34.9 g/dL
Red Cell Distribution Width (RDW)	14.3	14.4	13.4	12.4-17.3%
Platelet Count	251	116	196	150-400 ×10⁹/L
Mean Platelet Volume (MPV)	11.9	13.2	11.6	7.3-11.3 fL
International Normalised Ratio (INR)	2.84	–	1.5	0.8-1.2

**Figure 1:**
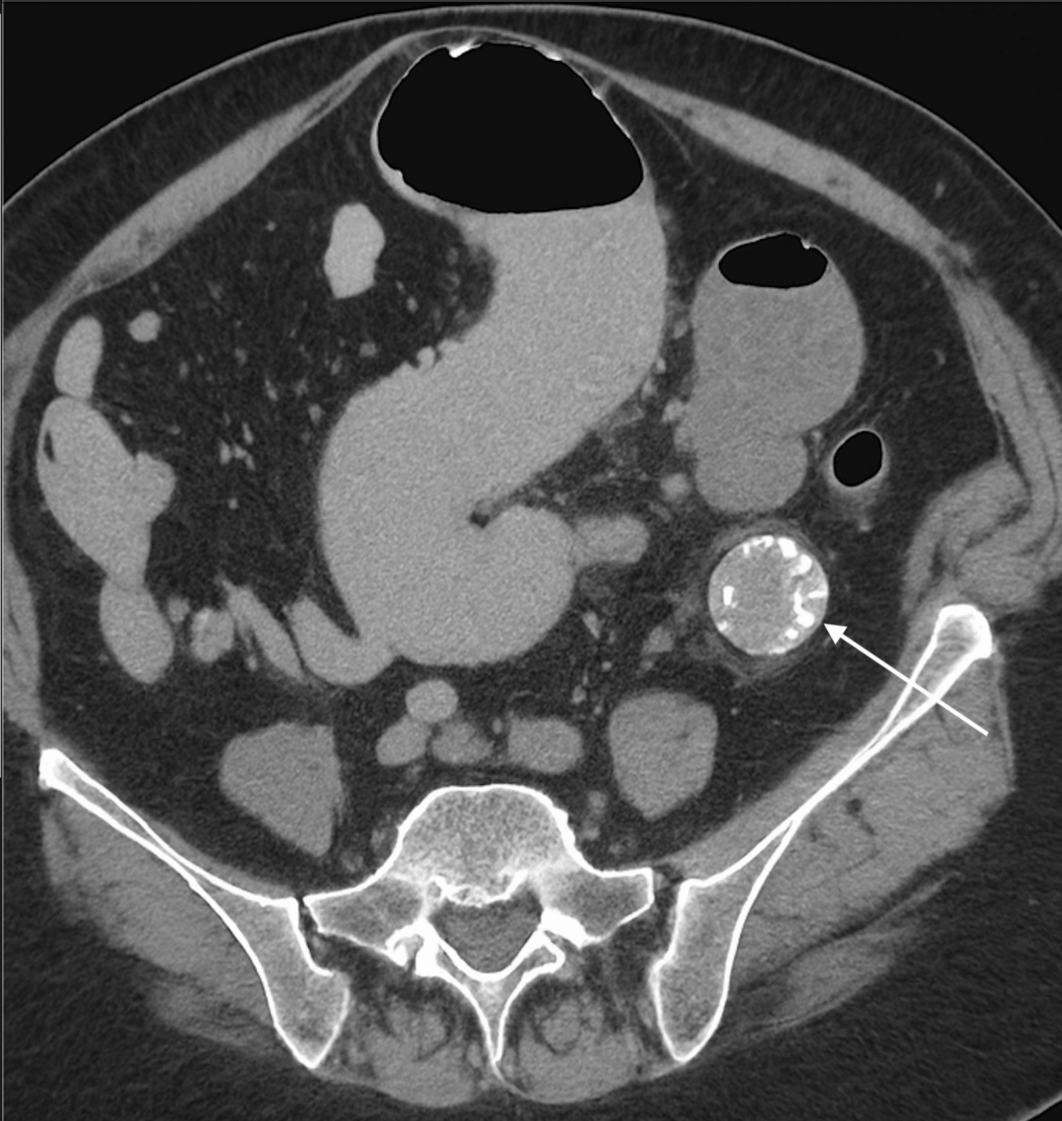
CT axial view showing ectopic gallstone (arrow). Axial CT image showing a hyperdense ectopic gallstone (white arrow) located in the distal jejunum/proximal ileum. Pneumobilia is also visible in the intrahepatic biliary tree. These findings support the diagnosis of gallstone ileus. Scale bar = 1 cm.

**Figure 2:**
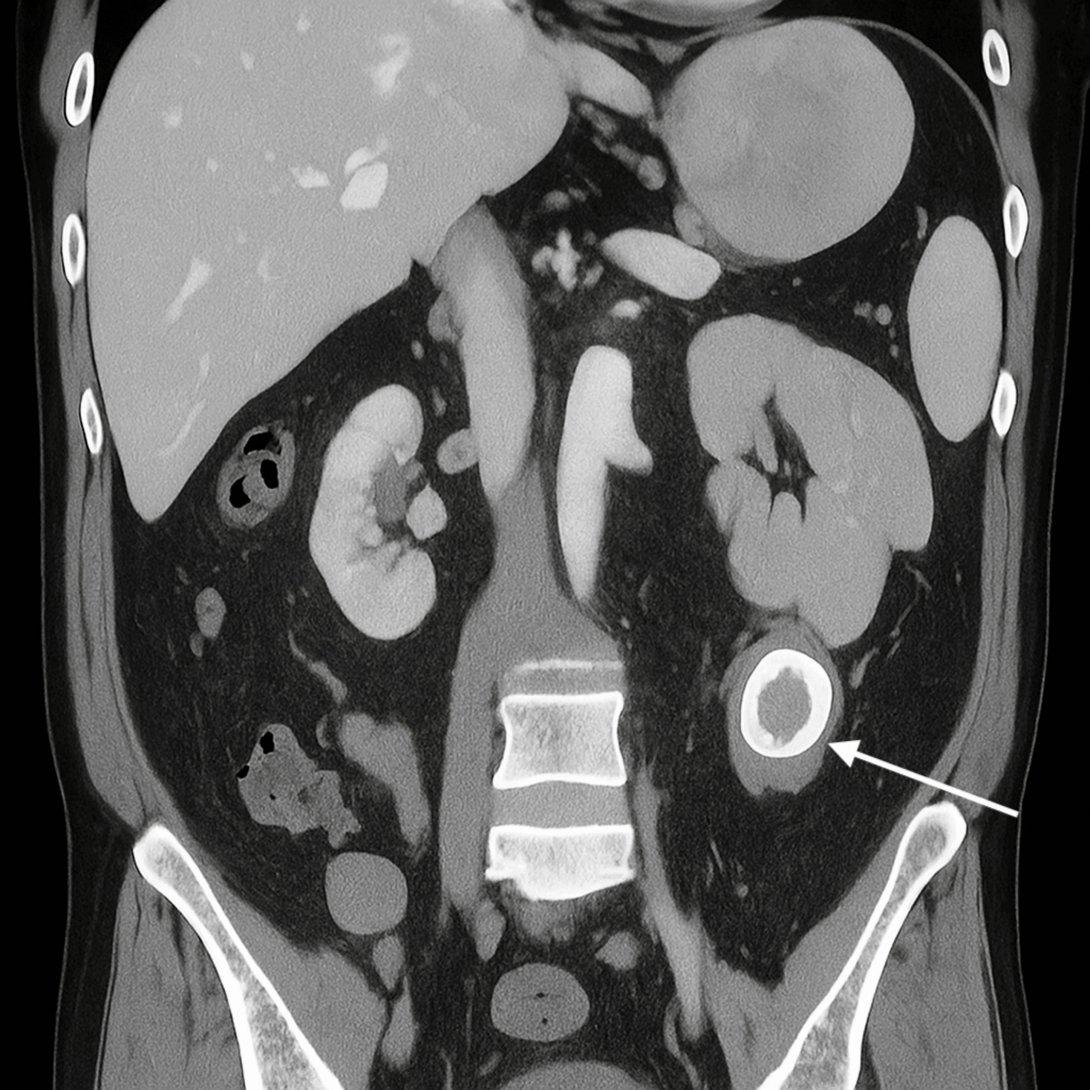
CT coronal view showing ectopic gallstone (arrow). Coronal CT image demonstrating the same ectopic gallstone (white arrow) with surrounding bowel dilatation. A cholecystoduodenal fistula is suspected based on adjacent wall thickening. Scale bar = 1 cm.

CT imaging revealed the nasogastric tube in the gastric fundus, thickening of the gallbladder wall and adjacent duodenum, and features suggestive of a cholecystoduodenal fistula. Hyperdense gallstones were identified in the cystic duct and proximal duodenum. The classic Rigler’s triad of pneumobilia, small bowel dilatation, and an ectopic gallstone was noted. The ectopic stone, measuring 43 × 27 mm, was located at the distal jejunum or proximal ileum, marking the transition point and confirming the diagnosis of gallstone ileus (Figures 1-2).

**Figure 3:**
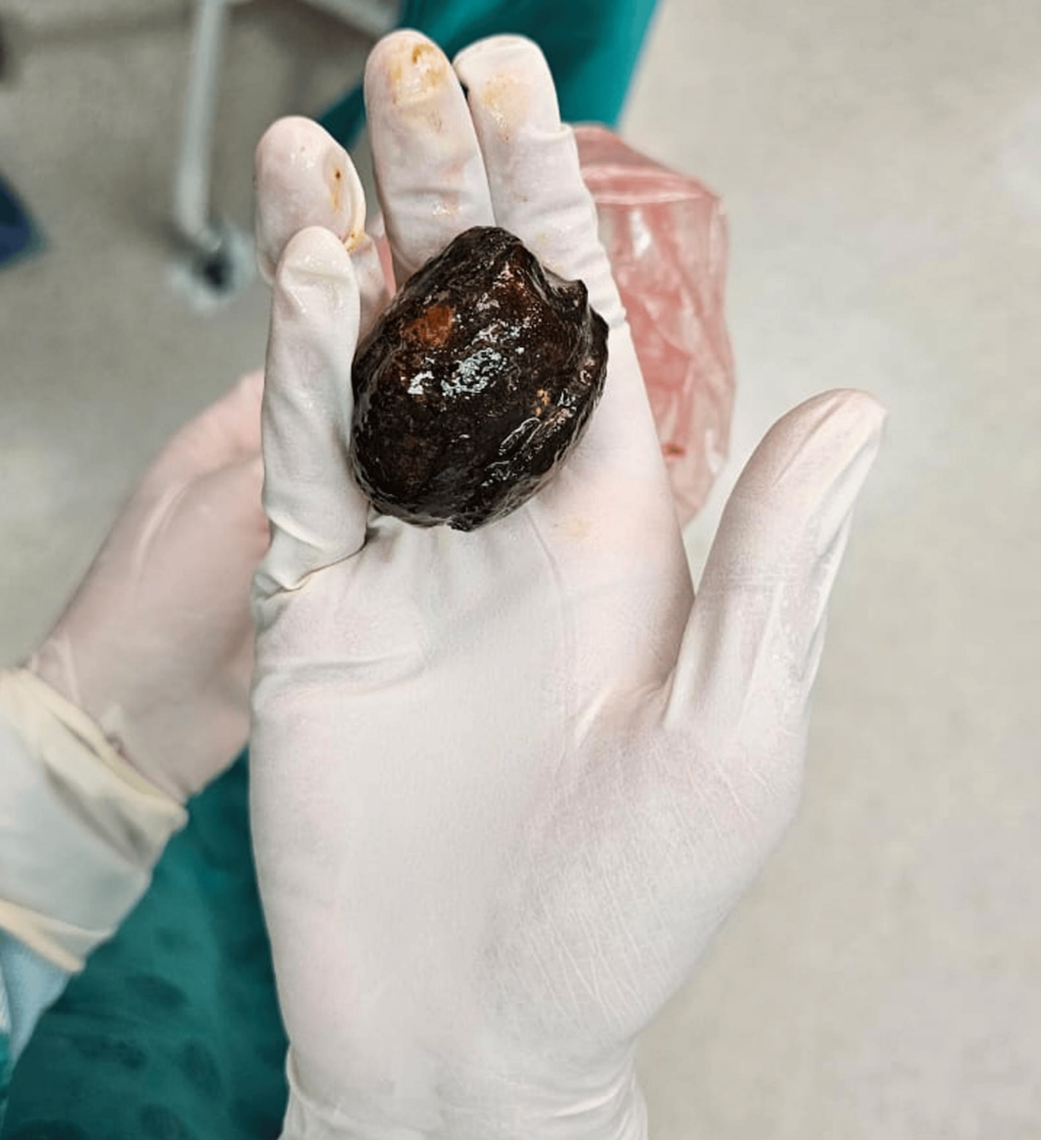
Gallstone retrieved intraoperatively. Intraoperative image of the retrieved gallstone, measuring approximately 5 × 5 cm. Its size and location confirmed it as the obstructive source in the ileum.

In light of these findings, the surgical team proceeded with an emergency exploratory laparotomy. Intraoperatively, a large gallstone approximately 5 × 5 cm in size was found impacted in the proximal ileum (Figure 3). An enterotomy was performed to extract the stone. The small bowel was closed in two layers using 3-0 polydioxanone suture (PDS) suture, the sheath was closed with loop PDS, and the abdominal wall was closed with 0 Vicryl in the subcutaneous layer and skin clips. No signs of bowel perforation or ischaemia were noted.

Postoperatively, the patient was monitored in a high-care setting. Her hospital stay was complicated by a superficial surgical site infection, which was managed with daily wound dressings using Betadine and Jelonet gauze. She made steady progress, with gradual reintroduction of oral intake and removal of both the nasogastric tube and urinary catheter. Once clinically stable, she was discharged and followed up at the surgical outpatient department for ongoing wound care and monitoring.

## Discussion

Gallstone ileus is a rare but serious complication of cholelithiasis, accounting for approximately 1-4% of all cases of mechanical small bowel obstruction, but up to 25% in elderly patients [6-8]. It results from the migration and impaction of one or more gallstones within the gastrointestinal tract, causing mechanical obstruction. The condition is often under-recognised due to its intermittent and non-specific symptoms, leading to diagnostic delays and, consequently, delayed treatment [6,7]. Several case reports have demonstrated that delayed presentation of gallstone ileus can result in considerable morbidity and prolonged hospital stays [9,10].

In elderly patients presenting with signs of small bowel obstruction, several differential diagnoses must be considered, including but not limited to: adhesions (especially in those with a history of prior abdominal surgery), incarcerated or strangulated hernias, small bowel tumours (such as adenocarcinoma and lymphoma), and volvulus [11]. In the present case, the absence of previous abdominal operations made adhesional obstruction less likely. Cross-sectional imaging excluded hernias and neoplastic lesions. The presence of pneumobilia and an ectopic calcified mass on CT imaging was highly suggestive of gallstone ileus, prompting surgical intervention.

The pathogenesis of gallstone ileus typically involves the formation of a cholecystoenteric fistula, most commonly between the gallbladder and the duodenum, through which gallstones enter the GI tract. This occurs due to chronic inflammation and pressure necrosis caused by large gallstones, leading to the development of adhesions and eventual fistulisation [2,11]. Approximately 60% of gallstones pass into the duodenum due to its close proximity to the gallbladder [12,13]. A less common but notable aetiology includes gallstone spillage during laparoscopic cholecystectomy, where retained stones may lead to abscess formation and eventual erosion into the bowel [12].

Diagnosing gallstone ileus can be particularly challenging. Laboratory results are usually non-specific. The classic radiographic features, collectively known as Rigler’s triad, include pneumobilia, signs of bowel obstruction, and an ectopic gallstone [6,7]. While plain abdominal radiographs may detect some or all of these features, their sensitivity ranges from 40-70% [1]. Ultrasound is often of limited value due to overlying bowel gas and difficulty in identifying either the stone or the fistula [2]. CT remains the gold standard for diagnosis, with a reported sensitivity of up to 93%. CT enables identification of the site of obstruction, the ectopic gallstone, and occasionally the fistulous tract itself [6-8,10]. Despite these advancements, many cases are still diagnosed intra-operatively, particularly when imaging findings are equivocal or unavailable [5].

Surgical management is aimed at relieving the obstruction and stabilising the patient. In elderly individuals, delayed diagnosis and intervention are associated with mortality rates as high as 12-27% [12]. The most commonly performed and widely accepted operation is simple enterolithotomy, which involves removal of the obstructing stone through a targeted bowel incision. This approach is typically favoured in elderly or high-risk patients due to its shorter operative time and reduced morbidity. In fit patients with minimal comorbidity and adequate preoperative stabilisation, a one-stage procedure, comprising enterolithotomy, cholecystectomy, and fistula closure, may be considered. However, this carries a higher risk of postoperative complications and should be reserved for selected cases. A two-stage approach, involving interval cholecystectomy after initial stone removal, is reserved for patients with persistent biliary symptoms or recurrent episodes following the initial procedure [7,14].

Endoscopic techniques have been described in selected cases, particularly when the gallstone is located in the stomach or duodenum. While endoscopic extraction may be feasible, it carries a risk of bleeding, and closure of any resulting enterotomy, if required, can be technically challenging in the context of inflamed or friable bowel [6,10]. Several case reports have documented successful endoscopic retrieval of duodenal gallstones; however, technical success remains limited to carefully selected patients [15,16]. Several case reports have also highlighted the successful use of endoscopic and minimally invasive techniques, including laser lithotripsy, in carefully selected patients with duodenal impaction or Bouveret’s syndrome [17-19].

In this case, the patient underwent a successful enterolithotomy. A decision was made not to proceed with fistula repair or cholecystectomy due to the high operative risk associated with her comorbidities. Her uneventful recovery and positive surgical outcome reflect the benefit of timely recognition and intervention in cases of gallstone ileus.

## Conclusions

Gallstone ileus is an uncommon but clinically significant cause of small bowel obstruction, particularly affecting the elderly population. Its non-specific and often intermittent presentation contributes to frequent delays in diagnosis and treatment. A high index of suspicion is therefore essential, especially in patients with a known history of cholelithiasis presenting with features of bowel obstruction. CT is the most effective imaging modality for confirming the diagnosis, with Rigler’s triad serving as a hallmark radiological finding.

Prompt surgical intervention, typically via enterolithotomy, remains the cornerstone of management and is associated with favourable outcomes when performed early. Although the obstructing stone was removed, the underlying biliary-enteric fistula was left in situ, which may pose a risk of recurrence and necessitates ongoing clinical follow-up. This case highlights the importance of considering gallstone ileus in the differential diagnosis of small bowel obstruction in elderly patients. Early imaging and timely surgical management are critical in reducing morbidity and improving clinical outcomes. Postoperative improvement in bilirubin and inflammatory markers further supported resolution of the obstruction and systemic inflammation.
